# Overestimation of the second time interval replaces time-shrinking when the difference between two adjacent time intervals increases

**DOI:** 10.3389/fnhum.2014.00281

**Published:** 2014-05-14

**Authors:** Yoshitaka Nakajima, Emi Hasuo, Miki Yamashita, Yuki Haraguchi

**Affiliations:** ^1^Department of Human Science, Research Center for Applied Perceptual Science, Kyushu UniversityFukuoka, Japan; ^2^Japan Society for the Promotion of Science/Neurological Institute, Kyushu UniversityFukuoka, Japan; ^3^Kyushu Institute of DesignFukuoka, Japan; ^4^Department of Acoustic Design, Kyushu UniversityFukuoka, Japan

**Keywords:** time perception, assimilation, contrast, audition, time-shrinking, empty interval

## Abstract

When the onsets of three successive sound bursts mark two adjacent time intervals, the second time interval can be underestimated when it is physically longer than the first time interval by up to 100 ms. This illusion, time-shrinking, is very stable when the first time interval is 200 ms or shorter (Nakajima et al., 2004, Perception, 33). Time-shrinking had been considered a kind of perceptual assimilation to make the first and the second time interval more similar to each other. Here we investigated whether the underestimation of the second time interval was replaced by an overestimation if the physical difference between the neighboring time intervals was too large for the assimilation to take place; this was a typical situation in which a perceptual contrast could be expected. Three experiments to measure the overestimation/underestimation of the second time interval by the method of adjustment were conducted. The first time interval was varied from 40 to 280 ms, and such overestimations indeed took place when the first time interval was 80–280 ms. The overestimations were robust when the second time interval was longer than the first time interval by 240 ms or more, and the magnitude of the overestimation was larger than 100 ms in some conditions. Thus, a perceptual contrast to replace time-shrinking was established. An additional experiment indicated that this contrast did not affect the perception of the first time interval substantially: The contrast in the present conditions seemed unilateral.

## INTRODUCTION

When the onsets of three successive sound bursts mark two neighboring time intervals, the second time interval can be underestimated when it is longer than the first time interval by up to 100 ms. This underestimation, i.e., time-shrinking, is very stable when the first time interval is 200 ms or shorter (Nakajima et al., 1991, 2004), and has been considered a kind of perceptual assimilation. Assimilation and contrast in perceptual paradigms often replace each other when the relationship and configuration of stimuli are changed systematically (e.g., Helson, 1963; Morinaga and Noguchi, 1966).

Assimilation and contrast may not necessarily be governed by a single perceptual mechanism, but they are likely to work under one perceptual principle for humans and animals to process information from the environment efficiently and quickly. For example, a figure in which luminance is sufficiently higher than in the background can be distinguished clearly from the background in the visual modality. This process is enhanced by contrast, which enlarges the perceptual difference in terms of lightness or color between the figure and the background, as well as by assimilation, which homogenizes the lightness or color within the figure and within the background (Koffka, 1935; Shapley and Reid, 1985). It is also argued that, when two potential objects are separated enough spatially from each other (but within a distance to keep a mutual interaction), they are likely to be organized as two separate wholes which are then contrasted (King, 1988). It is widely observed that perceptual assimilation between objects gives way to contrast when the difference between these objects is increased, and that assimilation can be blocked if the area or the group to be assimilated is broken by a boundary (or boundaries; e.g., Koffka, 1935; Hamburger, 2005), or by a temporal distance (Ikeda and Obonai, 1955). In Ikeda and Obonai’s (1955) experiment, concentric circles with different diameters *I* and *T* were presented simultaneously for 500 ms using a tachistoscope. The diameter of *T*, whose size was to be judged, was fixed at 30 mm. When the physical size of* I* was similar to that of *T*, assimilation took place, but contrast took over when the physical size difference was larger (**Table 1**). The fact that assimilation and contrast can both take place in the same experimental context is described systematically by Helson (1964). One should note that temporal configurations of stimuli can also lead to an assimilation or contrast of the stimuli (Shigeno, 1991; see also McKenna, 1984). In our study, assimilation and contrast were manipulated through modifying the temporal configuration of the sound bursts.

**Table 1:** Underestimation and overestimation of the size of a circle, *T* = 30 mm, caused by another concentric circle, *I*, as observed by Ikeda and Obonai (1955).

Diameter of *T*	30					
Diameter of* I*	10	15	20	40	60	80
Overestimation of* T*	+1.2	-0.7	-1.4	+0.6	+0.3	-0.5
	C	A	A	A	A	C

*The values are in millimeters. A, assimilation; C, contrast.*

When the difference between close but distinguishable objects or events is small, the objects will be seen as part of a homogeneous group. If the difference cannot be neglected, the objects or events will instead be perceived in different categories. This is the case particularly for the human auditory modality, which is responsible for quick and complicated communication sometimes in noisy environments without favorable acoustics.

Linguistic communication depends on the human capacity to process strings of categorized elements in time. This requires that any pair of sounds or sound patterns should be clearly either the same or different (de Saussure, 1966); assimilation and contrast must work for the listener to decode speech signals properly (e.g., Shigeno, 1991). Temporal aspects of auditory perception are also very likely to work in the same manner. Relative lengths of syllables are categorized in many languages; it is often important for the listener to judge, without hesitation, whether or not one of two neighboring syllables is longer or shorter than the other. When time intervals are presented in concatenation, listeners often simplify the patterns reducing small differences, and exaggerating larger differences (e.g., Fraisse, 1978, 1982; Povel, 1981). A ratio 1:2 or 2:1 seems stable perceptually, which means that the second time interval is likely to be overestimated if the neighboring time intervals are to be perceived as in a ratio 1:1.7 or 1:1.8 otherwise. We were interested in whether the extremely stable illusion of time-shrinking, a unilateral assimilation of a time interval to a preceding time interval or preceding time intervals, could be grasped in relation to such opposite perceptual processes. We thus examined whether a time interval was contrasted, instead of assimilated, to a preceding time interval at a certain point when the difference between these adjacent time intervals was increased step by step. When two adjacent empty time intervals *t*_P_ and *t*_S_ were presented in this order in our previous research, the same *t*_P_ may have caused both underestimation and overestimation of *t*_S_ depending on the physical difference between *t*_P_ and *t*_S_. Nakajima et al.’s (2004) experiments suggested that this possibility is systematic. **Table 2** indicates the cases in which both underestimation and overestimation reached 20 ms for a fixed *t*_P_ value.

**Table 2:** Temporal patterns in which time shrinking was replaced by overestimation in Nakajima et al. (2004).

Experiment 1

UE				OE	
| 160| 200|	| 160| 240|			| 160| 320|	| 160| 480|
**Experiment 2**

**UE**				**OE**	
| 160| 220|	| 160| 240|	| 160| 260|	| 160| 280|	| 160| 320|	
**Experiment 3**

**UE**				**OE**	
| 160| 200|	| 160| 240|			| 160| 320|	
| 200| 240|	| 200| 280|			| 200| 320|	| 200| 360|
| 240| 280|				| 240| 360|	| 240| 400|
**Experiment 4**

**UE**				**OE**	
| 280| 360|				| 280| 440|	
| 320| 400|				| 320| 480|	

*Both the underestimation of a standard time interval, i.e., time shrinking, and the overestimation of a longer standard appeared for the same preceding time interval in the stimulus conditions indicated in each line. The physical durations of two adjacent time intervals *t*_P_ and *t*_S_ are indicated as | *t*_P_| *t*_S_| in milliseconds. The conditions in which the underestimation/overestimation was equal to or above 20 ms were taken up to specify these temporal patterns. UE, underestimation; OE, overestimation.*

The present paradigm thus became clear. Time-shrinking typically takes place when two time intervals, *t*_P_ and *t*_S_ in this order, marked by the onsets of three successive sound bursts meet the following conditions: 0 < *t*_S_ - *t*_P_ ≤ 80 ms, and *t*_P_ ≤ 200 ms. It had been indicated already that overestimation of *t*_S_ to exaggerate the difference between *t*_P_ and *t*_S_ could take place when the physical difference between the neighboring time intervals, *t*_S_ - *t*_P_, exceeded the above range (Nakajima et al., 2004). This problem had never been taken up systematically. In order to reveal the mechanism of rhythmic organization, however, it seemed of crucial importance to examine whether a systematic overestimation of *t*_S_ would replace the underestimation, which we call time-shrinking, if we increased the difference *t*_S_ - *t*_P_.

## GENERAL METHODS

**FIGURE 1:**
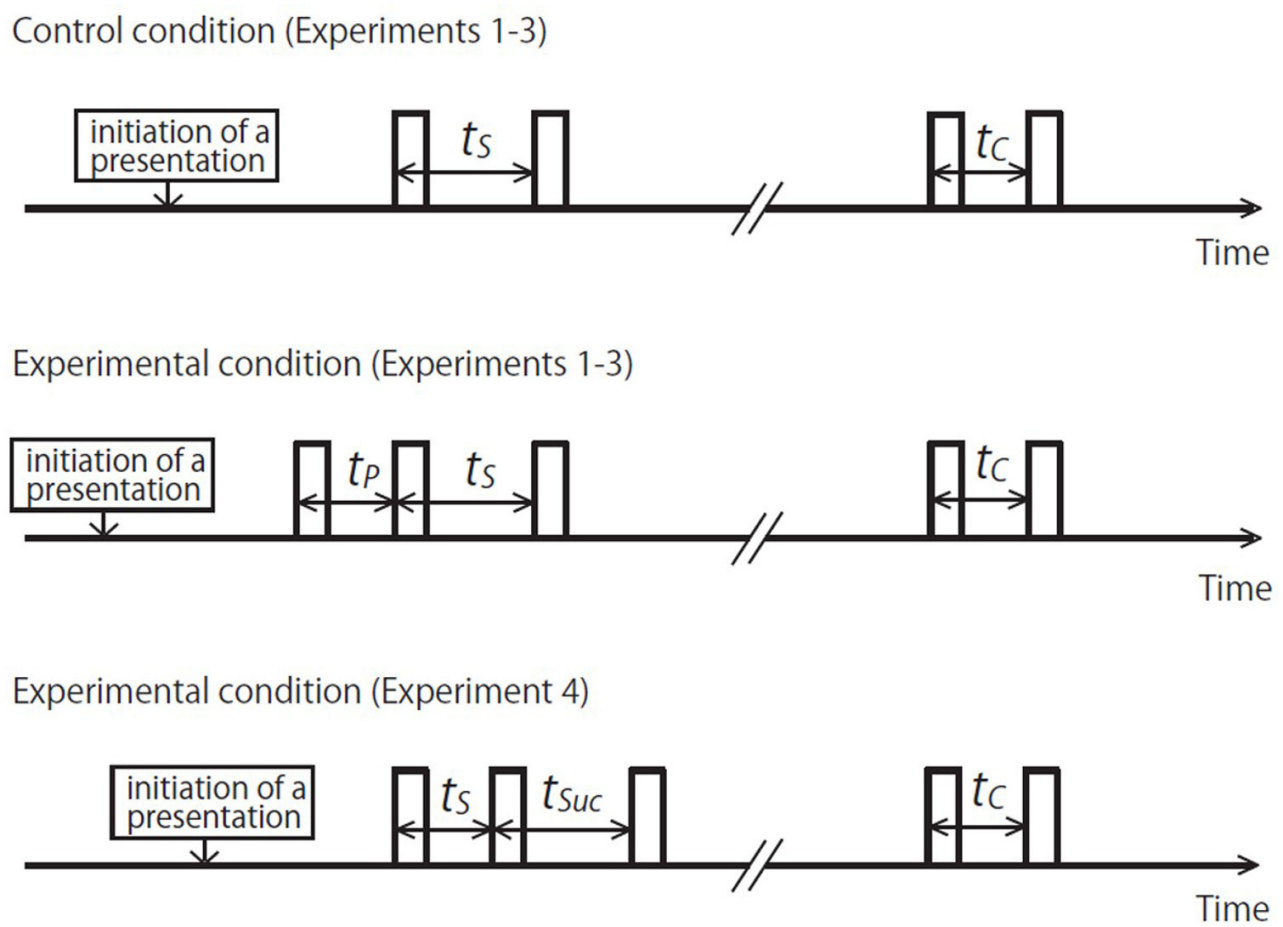
**Time charts of stimulus patterns.** The rectangles represent sounds. In the experiments, participants adjusted *t*_C_ to make its subjective duration equal to that of *t*_S_. In the experimental conditions of Experiments 1–3, *t*_P_ was added before *t*_S_. In the experimental condition of Experiment 4, *t*_SUC_ was added after *t*_S_. Note that all time intervals (*t*_S_, *t*_P_, *t*_SUC_, and *t*_C_) refer to the duration between the onsets of successive sounds.

The general framework common to the present experiments is described in **Figure 1**. In the first three experiments, we basically followed the paradigm employed in previous studies on time-shrinking (e.g., Nakajima et al., 2004), except that we increased the range of the standard duration to be judged. In the control condition, a time interval,* t*_S_, marked by the onsets of two successive tone bursts was the standard to be judged. An additional tone burst preceded *t*_S_ in the experimental condition; the effect of the preceding time interval, *t*_P_, marked by the onsets of this additional tone burst and the first marker of *t*_S_ was studied. The difference in subjective duration of *t*_S_ between the control and the experimental condition was measured.

In the last experiment, Experiment 4, a tone burst did not precede but succeeded *t*_S_, and the effect of the succeeding time interval, *t*_SUC_, marked by the onsets of the second marker of *t*_S_ and this additional tone burst was examined in order to interpret the results of the first three experiments. This was the experimental condition, and no control condition was employed because the data of the control condition in Experiment 3 could be reused.

The method of adjustment was employed. The participant initiated each presentation by clicking a pane on the computer screen. A few seconds – the interval was chosen randomly within a range – after the clicking, the first tone burst of the standard pattern *t*_S_, *t*_P_|*t*_S_, or *t*_S_| *t*_SUC_ was presented. After that, there was a period of a few seconds – the interval was again chosen randomly, and then, another time interval, the comparison, *t*_C_, was presented with the onsets of two successive tone bursts. The task of the participant was to adjust *t*_C_ to make it equal to *t*_S_ in subjective duration. The participant could change *t*_C_ by operating a screen interface, designed in a way not to give a visual hint about the present duration, and the minimum step of the adjustment was 1 ms. The participant was allowed to listen to the whole sequence as many times as he/she needed until *t*_S_ and *t*_C_ were perceived as equal, and finished the trial when satisfied. The last *t*_C_ value was recorded as the point of subjective equality, PSE.

## EXPERIMENT 1

This experiment was conducted in 1996. Because we did not have an institutional ethical committee for psychological experiments at that time, an internal ethical review was impossible, but the experiment was a part of a research project reviewed by a governmental committee to select projects to be funded (as in the acknowledgments). This experiment is included in the present report because this was the first case in which the perceptual phenomenon we are going to describe appeared systematically. Our original purpose had been to determine the stimulus conditions to investigate the effect of sound marker duration on the occurrence of time-shrinking (underestimation), for there was a possibility that the amount of time-shrinking may be reduced, or the time condition for maximum time-shrinking could be shifted, by lengthening the markers (see Hasuo et al., 2011). From the present viewpoint, however, the experimental data gave us insight into the possibility of systematic overestimation of the second of two adjacent time intervals. The same *t*_S_ values were employed with a *t*_P_ in the experimental condition and in isolation in the control condition. The PSEs in these conditions were compared to see the amount of perceptual overestimation or underestimation of *t*_S_ caused by *t*_P_.

### METHODS

#### Participants

The participants were five students, i.e., three males and two females, of the Kyushu Institute of Design (the predecessor of the Faculty of Design, Kyushu University). They had received education for acoustic design, including basic training in music performance. They were 20–24 years old, and had normal hearing.

#### Materials

Duration markers were pure tone bursts of 1000 Hz and 12, 63, or 123 ms with a rise and a fall time of ~2 ms each. These values were inexact due to our use of an analog filter to shape the waveform; the inexactness was sufficiently small relative to the effect we were measuring. The tone bursts of different durations were approximately equal in loudness when presented separately. This was realized by conducting preliminary measurements in which the participant could listen to any of the three sounds by clicking corresponding buttons on the computer screen. The stimulus sound was presented always 200 ms after the button was clicked. The level of the 12-ms burst, which was very short, was fixed at 97 dBA as defined as the level of a continuous tone of the same amplitude measured with an artificial ear (Brüel and Kjær 4153), a microphone (Brüel and Kjær 4134), and a sound level meter (Brüel and Kjær 2209). The levels of the other sounds were adjustable, and the participant was instructed to equalize the three sounds in terms of loudness. In each trial, the adjusted levels of the 63 and 123 ms bursts were recorded. The participant performed eight trials, and the median value for each sound was employed as the presentation level in the main part of the experiment. The presentation levels were 87–94 dBA for the 63-ms burst, and 85–93 dBA for the 123-ms burst.

The pure tones were first generated as rectangular pulse series before being band-pass filtered between 850 and 1250 Hz (NF DV-6BW). This resulted in tone bursts with rise and fall times of ~2 ms. The tone bursts were presented to the left ear of the participant through an amplifier (JVC AX-Z511) and headphones (AKG K141) in a soundproof room. The experimental procedure including stimulus generation was controlled by a quiet computer without a hard disk drive or a fan (Commodore Amiga 500).

In the main part of the experiment, the marker duration was fixed in each standard pattern, which was marked by two or three successive tone bursts, and the comparison time interval was always marked by two 12-ms tone bursts. In the standard patterns of the experimental condition, *t*_P_| *t*_S_, the preceding time interval, *t*_P_, was fixed at 160 ms. Both in the control and in the experimental condition, the standard time interval, *t*_S_, was varied from 120 to 440 ms in steps of 40 ms. The *t*_S_ duration of 120 ms was not possible when the marker duration was longer, i.e., 123 ms; this condition was omitted. Thus, there were 58 stimulus patterns: [2 (control/experimental) × 2 (marker durations ≤ 63 ms) × 10 (*t*_S_ durations) + 1 (marker duration = 123 ms) × 9 (*t*_S_ durations)]. The standard pattern was presented 2300–2500 ms after the participant clicked a button on the screen. There was a silence of 2700–3300 ms between the offset of the last sound marker of *t*_S_, and the onset of the first sound marker of *t*_C_.

#### Procedure

The participant performed four adjustment trials, two in ascending series and two in descending series, for each stimulus pattern: two replications for both series were performed. One replication comprised the first half, and the other the second half of the whole measurement. Each replication (= half) consisted of 116 trials, 58 (stimulus patterns) × 2 (series) in random order, and was divided into 9 blocks of 12 or 13 measurement trials, which were preceded by two warm-up trials. Preceding the measurement, the participant performed 58 training trials, divided into four blocks; each stimulus pattern appeared once. Thus, the whole experiment consisted of 22 blocks: 4 (training blocks) + 2 (replications) x 9 (measurement blocks). Each block took around 15–20 min, and the whole experiment was carried out over a period of 8 days for each participant.

### RESULTS AND DISCUSSION

We performed a three-way [marker duration × condition (experimental/control) × *t*_S_ duration] ANOVA utilizing the PSEs for *t*_S_ = 160–480 ms. Since it is commonplace that PSEs change as *t*_S_ changes, we will not detail the main effect of this factor neither here nor in the following experiments; its main effect was always significant (*p* < 0.001). The main effect of marker duration was significant, *F*(2,8) = 21.902, *p* < 0.01, $\eta_{p}^{2}$ = 0.846. Ryan’s *post hoc* test showed that the difference between all combinations of marker duration, i.e., 12 and 123; 63 and 123; and 12 and 63 ms; was significant (*p* < 0.05). The interaction between condition (experimental/control) and *t*_S_ duration was also significant, *F*(8,32) = 4.614, *p* < 0.01, $\eta_{p}^{2}$ = 0.536. This interaction should be related to the assimilation and contrast of *t*_S_ to *t*_P_. The main effect of condition (experimental/control) and the other interactions were not significant (*p* > 0.05).

**FIGURE 2:**
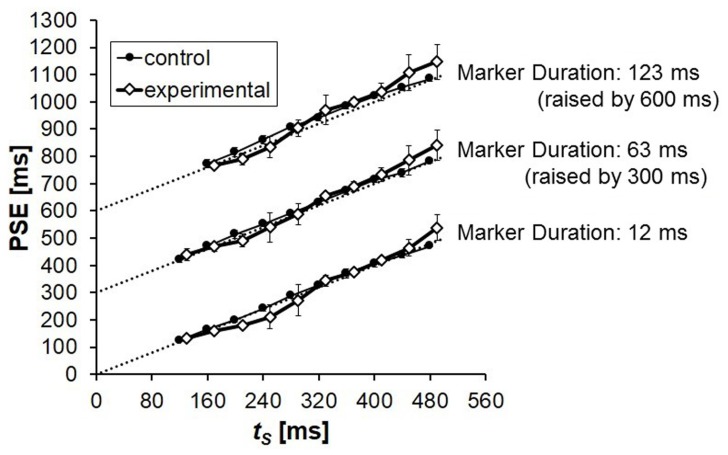
**Mean PSEs obtained from five participants in Experiment 1.** PSE corresponds to the duration of *t*_C_ that was perceived to be equal to the duration of *t*_S_. The results for marker durations 63 and 123 ms were raised by 300 and 600 ms, respectively, in this graph for clarity. The physical values of *t*_S_ (the points of objective equality) are indicated by dotted lines. Error bars represent standard deviations between participants.

The PSEs in the control condition were very close to the physical values of *t*_S_ (**Figure 2**). Slight deviations appeared systematically, however: PSEs of shorter duration tended to be longer than the physical values of *t*_S_. This kind of time errors sometimes appear in the literature of time perception (Woodrow, 1951; Eisler et al., 2008). The PSEs tended to be slightly longer when the marker duration was longer, but the present data do not offer much information on this issue. This issue should be investigated intensively in the future in order to understand rhythm perception in speech or music. Hasuo et al. (2011, 2012) reported that inter-onset time intervals up to 360 ms tended to be perceived as longer when the duration of the sound markers to terminate the time intervals were longer. This was the case whether the time interval to be judged was isolated or neighboring another time interval. The duration of the sound markers to initiate the time intervals showed similar effects, but in a more unstable manner.

The PSEs in the control and in the experimental condition differed systematically. The experimental PSEs were smaller than the corresponding control PSEs when *t*_S_ = 200 or 240 ms, i.e., when *t*_S_ - *t*_P_ = 40 or 80 ms: *t*_S_ was underestimated showing time-shrinking in a typical manner. However, the difference between the control and the experimental condition was reversed when *t*_S_ was longer: the experimental PSEs were systematically greater than the control PSEs when *t*_S_ ≥ 320 ms. Thus, time-shrinking as assimilation of *t*_S_ to *t*_P_ appeared when the difference between these neighboring time intervals was small, and gave way to contrast of *t*_S_ to *t*_P_ when the difference was large.

The above tendency appeared in similar ways in all the marker conditions between the control and the experimental PSEs despite the fact that the control PSEs increased slightly, but clearly, if the sound marker duration was increased. The contrast appeared as overestimation of *t*_S_ in the experimental condition against the control condition. The PSEs were already lengthened in the control condition if the sound markers were longer, and they became even longer – were overestimated further – in the experimental condition. Furthermore, the amount of overestimation was larger when the duration markers were longer. This is in contrast with the fact that the magnitude of time-shrinking – underestimation – is often smaller when longer markers are used (Yamashita and Nakajima, 1999; Hasuo et al., 2011), as was the case also in the present experiment.

The overestimation, as represented by the difference in the PSEs between the control and the experimental condition, seemed to have a local peak when *t*_S_ = 320 ms for all the marker durations. This tendency was peculiar and robust, but we leave this issue for future research.

To test whether the common tendency in overestimation pattern (i.e., the difference between the control and the experimental PSEs over the *t*_S_ duration range) across different marker durations was statistically significant, we conducted a Friedman test (e.g., Siegel and Castellan, 1988) utilizing the mean overestimation values for each marker duration. There was a statistically significant tendency in overestimation, χ^2^(8) = 23.644, *p* = 0.003. To examine whether the overestimation patterns had a common tendency even when the influence of time-shrinking (the negative overestimation at *t*_S_ - *t*_P_ = 40 or 80 ms ) was cancelled, we also performed the same Friedman test without the conditions in which *t*_S_ - *t*_P_ = 40 or 80 ms. The tendency in overestimation pattern was significant again, χ^2^(6) = 17.714, *p* = 0.007. The statistical significance in this additional Friedman test confirmed that the overestimation patterns had a common tendency even without the influence of time-shrinking.

## EXPERIMENT 2

Experiments 2–4 were part of a research project approved by the research ethics committee of the Faculty of Design, Kyushu University, in 2010. Experiment 1 and our previous data on time-shrinking (e.g., Nakajima et al., 2004) revealed that the underestimation of a time interval that appeared as assimilation of *t*_S_ to *t*_P_ often gave way to contrast when* t*_S_ - *t*_P_ > 120 ms. Because we did not have systematic data indicating this effect except in Experiment 1, we decided to conduct an experiment in which *t*_S_ was varied in a larger range (up to 640 ms). For *t*_P_, we chose three values: 80, 120, and 160 ms. Time-shrinking appears most stably in this range of *t*_P_ (Nakajima et al., 2004; Miyauchi and Nakajima, 2005), and we first needed experimental data under such conditions. One of the things we were interested in was whether any overestimation would appear for *t*_P_ = 120 ms; there had been occasional cases in previous data in which *t*_S_ had been overestimated for *t*_P_ = 80 or 160 ms, but no such cases ever for *t*_P_ = 120 ms. Most importantly, we wanted to see whether the typical time-shrinking, which was expected reliably if *t*_S_ - *t*_P_ = 40 or 80 ms, would give way to contrast, i.e., overestimation of *t*_S_.

### METHODS

#### Participants

Five students of Kyushu University, three males and two females, participated. One of them had been educated to become a high-school music teacher, and three of them had received education for acoustic design, including basic training in music performance. The fifth one was an amateur musician who had been playing percussions for 8 years. They were 21–46 years old.

#### Materials

Duration markers were pure tone bursts of 1000 Hz and 10 ms with cosine-shaped rise and fall times of 5 ms each, with no steady-state part. Their level was 80 dBA as defined as the level of a continuous tone of the same amplitude measured with an artificial ear (Brüel and Kjær 4153), and a sound level meter (Node 2072 or 2075). The tone bursts were presented diotically to the participant through an amplifier (Stax SRM-323A) and headphones (Stax SR-303) in a soundproof room. The experimental procedure including stimulus generation was controlled by a computer (Frontier KZFM71/N) with an audio processor (Onkyo Wavio SE-U55GX). Stimulus patterns were generated digitally (16 bits; a sampling frequency of 44 100 Hz), and went through a 16-kHz low-pass filter (NF DV-8FL) to avoid aliasing.

In the standard patterns of the experimental condition, *t*_P_| *t*_S_, the preceding time interval, *t*_P_, was 80, 120, or 160 ms, for which time-shrinking had occurred typically in previous studies (e.g., Nakajima et al., 2004). Overestimation of *t*_S_ had been recorded for *t*_P_ = 80 and 160 ms, but only in a few stimulus patterns for each *t*_P_ value, and only up to 30 ms, except for Experiment 1 of the present article. For *t*_P_ = 120 ms, no related measurements had been done before. The standard time interval, *t*_S_, was varied from 40 to 640 ms in steps of 40 ms both in the experimental and in the control condition. There were 64 stimulus patterns: 4 (1 control + 3 *t*_P_ durations) × 16 (*t*_S_ durations). The standard pattern was presented 1500–2500 ms after the participant initiated a presentation. There was an interval of 3000–4000 ms between the onsets of *t*_S_ and *t*_C_.

#### Procedure

The participant performed two adjustment trials, one in ascending series and one in descending series, for each stimulus pattern, and thus 128 trials in total: 64 (stimulus patterns) × 2 (series), which were arranged in random order and divided into 11 blocks of 11 or 12 measurement trials preceded by two warm-up trials. Before the measurement, the participant performed one training session of 16 trials, for which representative stimulus patterns were employed. Thus, the whole experiment consisted of 12 blocks: 1 (training block) + 11 (measurement blocks). Each block took around 15–30 min, and the whole experiment was carried out over a period of 2–3 days for each participant.

### RESULTS AND DISCUSSION

We performed a two-way [condition (1 control + 3 *t*_P_ durations) × *t*_S_ duration] ANOVA utilizing the PSE values. The main effect of condition (1 control + 3 *t*_P_ durations) was significant, *F*(3,12) = 8.624, *p* < 0.01, $\eta_{p}^{2}$ = 0.683, and so was the interaction between condition (1 control + 3 *t*_P_ durations) and *t*_S_ duration, *F*(45,180) = 3.344, *p* < 0.01, $\eta_{p}^{2}$ = 0.455.

**FIGURE 3:**
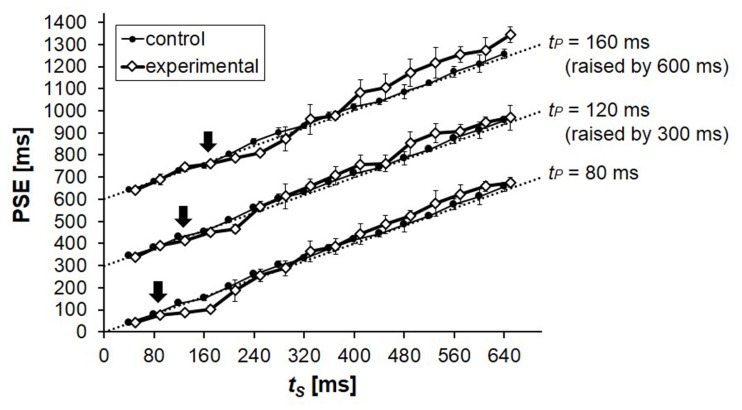
**Mean PSEs obtained from five participants in Experiment 2.** PSE corresponds to the duration of *t*_C_ that was perceived to be equal to the duration of *t*_S_. The results for *t*_P_ = 120 and 160 ms were raised by 300 and 600 ms, respectively, in this graph for clarity. The physical values of *t*_S_ (the points of objective equality) are indicated by dotted lines, on which *t*_P_ values are indicated by arrows. Error bars represent standard deviations between participants.

The PSEs in the control condition were close to the physical values of *t*_S_, but slight deviations appeared systematically (**Figure 3**). PSEs of longer duration tended to be longer than the physical values of *t*_S_, and this was not consistent with the tendency observed in Experiment 1. In both cases, however, the observed deviations were extremely small, and can be neglected for our present purpose.

The PSEs in the control and in the experimental condition differed systematically. The experimental PSEs were smaller when *t*_S_ = *t*_P_ + 40 or *t*_P_ + 80 ms, indicating a robust occurrence of time-shrinking. This underestimation of *t*_S_, however, was replaced by overestimation, whose highest magnitude reached above 50 ms, when *t*_S_ ≥ *t*_P_ + 240 ms for all the *t*_P_ values. Thus, as in Experiment 1, time-shrinking appeared when the difference between *t*_S_ and *t*_P_ was 40 or 80 ms, and contrast of *t*_S_ to *t*_P_ took over when *t*_S_ was lengthened.

When *t*_P_ = 160 ms as in Experiment 1, the overestimation again seemed to have a local peak when *t*_S_ = 320 ms. This tendency indeed seems interesting, but is an issue to be investigated in the future.

To test whether the common tendency in overestimation pattern across different *t*_P_ values (i.e., the underestimation of *t*_S_ when *t*_S_ = *t*_P_ + 40 or *t*_P_ + 80 ms and the overestimation when *t*_S_ ≥ *t*_P_ + 240 ms, observed for all *t*_P_ values) was statistically significant, we conducted a Friedman test utilizing the mean overestimation values for each *t*_P_ duration (= 80, 120, or 160 ms). There was a statistically significant tendency in overestimation depending on the difference between the two neighboring intervals (*t*_S_ - *t*_P_ = -40 to 480 ms), χ^2^(13) = 34.505, *p* = 0.001. As in Experiment 1, we also performed the same Friedman test without the conditions in which *t*_S_ - *t*_P_ = 40 or 80 ms, where time-shrinking should have taken place. The tendency in overestimation pattern was significant again, χ^2^(11) = 27.410, *p* = 0.004.

## EXPERIMENT 3

Time-shrinking almost disappeared, although not completely, when *t*_P_ was above 300 ms (Nakajima et al., 2004, Figure 11). Our next step was to examine whether the tendency for *t*_S_ to be underestimated when *t*_S_ = *t*_P_ + 40 or *t*_P_ + 80 ms and overestimated when *t*_S_ was further lengthened, as observed in Experiments 1 and 2, would appear entirely in the *t*_P_ range in which we could expect time-shrinking. Because the overestimation of *t*_S_ appeared in a very wide range of *t*_S_ in Experiment 2, we made the range of *t*_S_ in the present experiment even wider.

### METHODS

#### Participants

Six students of Kyushu University, three males and three females, participated. Four of them had taken part in Experiment 2, but there had been an interval of at least 3 months. One of the participants had been educated to become a high-school music teacher, and four of them had received education for acoustic design, including basic training in music performance. The sixth one was an amateur musician who had been playing percussions for 8 years. They were 20–46 years old.

#### Materials

Duration markers and the way of presentation were the same as in Experiment 2. In the standard patterns of the experimental condition, *t*_P_| *t*_S_, *t*_P_ = 40, 120, 200, or 280 ms, where time-shrinking had occurred clearly (Nakajima et al., 2004). Overestimation of *t*_S_ had been recorded for these *t*_P_ values, but only in a handful of stimulus patterns, and only up to 30 ms, except for Experiment 2 of the present article. The standard time interval, *t*_S_, was varied from 40 to 1000 ms in steps of 80 ms both in the control and in the experimental condition. There were 65 stimulus patterns: 5 (1 control + 4 *t*_P_ durations) × 13 (*t*_S_ durations). The standard pattern was presented 1500–2500 ms after the participant initiated a presentation. There was an interval of 4000–5000 ms between the onsets of *t*_S_ and *t*_C_.

#### Procedure

The participant performed two adjustment trials, one in ascending series and one in descending series, for each stimulus pattern, and thus 130 trials in total: 65 (stimulus patterns) × 2 (series), which were arranged in random order and divided into 10 blocks of 13 measurement trials preceded by two warm-up trials. Before the measurement, the participant performed 15 training trials, for which representative stimulus patterns were employed. Thus, the whole experiment consisted of 14 blocks: 1 (training block) + 13 (measurement blocks). Each block took around 15–30 min, and the whole experiment was carried out over a period of 2–3 days for each participant.

### RESULTS AND DISCUSSION

We performed a two-way [condition (1 control + 4 *t*_P_ durations) × *t*_S_ duration] ANOVA utilizing the PSE values. The main effect of condition (1 control + 4 *t*_P_ durations) was significant, *F*(4,20) = 6.450, *p* < 0.01, $\eta_{p}^{2}$ = 0.563, and so was the interaction between condition (1 control + 4 *t*_P_ durations) and *t*_S_ duration, *F*(48,240) = 2.539, *p* < 0.01, $\eta_{p}^{2}$ = 0.337.

**FIGURE 4:**
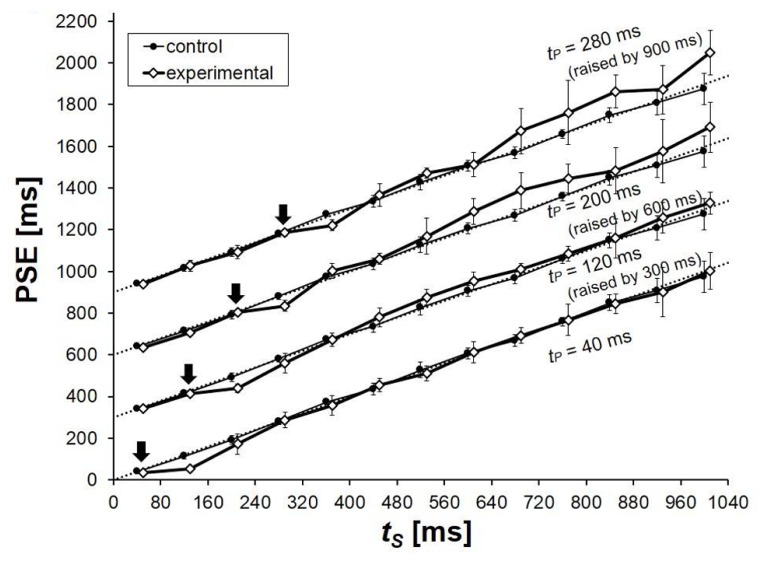
**Mean PSEs obtained from six participants in Experiment 3.** PSE corresponds to the duration of *t*_C_ that was perceived to be equal to the duration of *t*_S_. The results for *t*_P_ = 120, 200, and 280 ms were raised by 300, 600, and 900 ms, respectively, in this graph for clarity. The physical values of *t*_S_ (the points of objective equality) are indicated by dotted lines, on which *t*_P_ values are indicated by arrows. Error bars represent standard deviations between participants.

The PSEs in the control condition were very close to the physical values of *t*_S_ (**Figure 4**). Although slight deviations appeared again systematically, they were almost unrecognizable in the graphs except for the longest *t*_S_ values, for which PSEs tended to be slightly shorter than the corresponding points of objective equality.

The PSEs in the control and in the experimental condition differed systematically. The experimental PSEs were conspicuously smaller when *t*_S_ = *t*_P_ + 80 ms, again showing the robustness of time-shrinking. For *t*_P_ = 120, 200, and 280 ms, the underestimation of *t*_S_ was replaced by overestimation when *t*_S_ was longer. When *t*_S_ > *t*_P_ + 240 ms, the PSEs in the experimental condition were never smaller than those in the control condition. For *t*_P_ = 200 and 280 ms, the overestimation reached above 100 ms, which is comparable to the temporal illusions Israeli (1930) reported in the visual modality. For *t*_P_ = 40 ms, no clear overestimation appeared. When the same preceding interval duration was employed in Nakajima et al.’s (2004) Experiment 1, however, some overestimation appeared stably, although the amount was only about 10 ms, and it would be safer to reserve any clear conclusion for this *t*_P_ value. In the present experiment, time-shrinking appeared when the difference between *t*_S_ and *t*_P_ was 80 ms, and contrast of *t*_S_ to *t*_P_ took over when *t*_S_ was lengthened except when *t*_P_ = 40 ms.

As in Experiment 2, we conducted a Friedman test utilizing the mean overestimation values for each *t*_P_ duration to examine whether the common tendency in the overestimation pattern across different *t*_P_ values 40, 120, 200, and 280 ms was statistically significant. There was a statistically significant tendency in overestimation depending on the difference between the two neighboring intervals (*t*_S_ - *t*_P_ = 0–720 ms), χ^2^(9) = 25.855, *p* = 0.002. We also performed the same Friedman test, but without the (negative) overestimations in conditions in which *t*_S_ - *t*_P_ = 80 ms, where time-shrinking should have taken place. The tendency in overestimation pattern was significant again, χ^2^(8) = 19.600, *p* = 0.012, confirming that the overestimation patterns had a common tendency even when the influence of time-shrinking (the dip at *t*_S_ - *t*_P_ = 80 ms) was cancelled.

## EXPERIMENT 4

The overestimation of *t*_S_ took place to a remarkable degree in Experiments 1–3. It seemed necessary to have some idea on whether this strong contrast, which was observed between the two neighboring time intervals, *t*_1_ and *t*_2_ in this order, for the perception of *t*_2_, also affected the perception of *t*_1_. Because time-shrinking was a unilateral illusion affecting mainly the perception of *t*_2_, we first examined whether, and if so how, the underestimation of *t*_2_ gave way to overestimation, and this indeed happened to a remarkable degree. Now it seemed important to check whether this contrast was unilateral or bilateral. In the present study, we just conducted an experiment to be appended to Experiment 3, but this would help us to interpret the present results. We picked up six temporal patterns of two neighboring time intervals in which contrast between them had caused overestimation of *t*_2_ (*t*_S_ in Experiment 3). Then PSEs of *t*_1_ were measured for these patterns. For example, we took up a pattern of *t*_1_ = 200 ms and *t*_2_ = 680 ms, in which *t*_2_ had been overestimated by more than 100 ms in Experiment 3. In the present experiment, we were interested in whether or not the same mechanism of contrast (bilaterally) led to the underestimation of *t*_1_ making its PSE shorter than the control value. Because *t*_1_ was the standard time interval, it is called *t*_S_, and the succeeding time interval *t*_2_ is called *t*_SUC_ in the present report. In other words, we used the same temporal patterns of two neighboring time intervals marked by three successive sounds as in Experiment 3, and the key difference was that *t*_C_ was adjusted to match the perceived duration of the first interval instead of that of the second interval.

Due to the unavailability of a certain potential participant, we decided to employ five of the six participants from Experiment 3, making it still possible to reuse the data in the control condition of Experiment 3.

### METHODS

#### Participants

Five students, three males and two females, participated in this experiment after participating in Experiment 3. There had been an interval of at least 1 month between these experiments. They were 21–25 years old. Four of them had taken part in Experiment 2, but there had been an interval of at least 3 months. Four of them had received education for acoustic design, including basic training in music performance. The fifth one was an amateur musician who had been playing percussions for 8 years.

#### Materials

Six stimulus patterns were chosen from the stimulus patterns in Experiment 3. In the standard patterns of the experimental condition, *t*_S_| *t*_SUC_, the standard time interval, *t*_S_, was 120, 200, or 280 ms; these values had been chosen for *t*_P_ in Experiment 3. The control patterns of these *t*_S_ values in Experiment 3 were regarded as the virtual control patterns of the present experiment, and thus the control data of the present participants were reused. The succeeding time interval, *t*_SUC_, was 440 or 680 ms; *t*_SUC_ in any stimulus pattern would have been overestimated stably if it had been the standard time interval. There were six stimulus patterns not including the virtual control patterns. The standard pattern was presented 1500–2500 ms after the participant initiated a presentation. There was a silence of 4000–5000 ms between the onsets of *t*_S_ and *t*_C_.

#### Procedure

The participant performed two adjustment trials, one in ascending series and one in descending series, for each stimulus pattern, and thus 12 trials in total arranged in random order. Four trials were conducted first for training and a warm-up, and the measurement trials followed without a break. The experiment took around 20 min.

### RESULTS AND DISCUSSION

We performed a two-way [condition (1 control + 2 *t*_SUC_ durations) × *t*_S_ duration] ANOVA utilizing the PSE values. Neither the main effect of condition (1 control + 2 *t*_SUC_ durations) nor the interaction between condition (1 control + 2 *t*_SUC_ durations) and *t*_S_ duration was significant, *F*(2,8) = 0.222, *p* > 0.05, $\eta_{p}^{2}$ = 0.052; *F*(4,16) = 2.740, *p* > 0.05, $\eta_{p}^{2}$ = 0.407, respectively.

**FIGURE 5:**
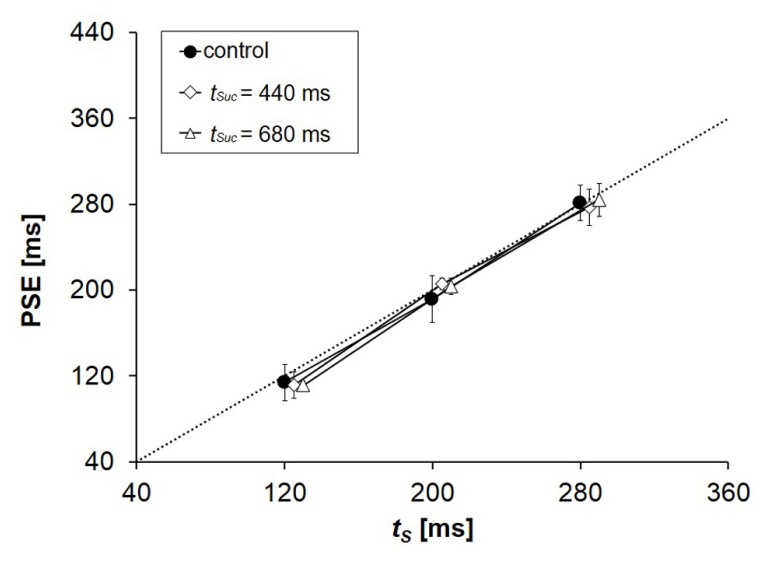
**Mean PSEs obtained from five participants in Experiment 4.** PSE corresponds to the duration of *t*_C_ that was perceived to be equal to the duration of *t*_S_. Some dots are deviated slightly from the scale marks on the horizontal axis to avoid being invisible in the graph. The physical values of *t*_S_ (the points of objective equality) are indicated by a dotted line. Error bars represent standard deviations between participants.

The PSEs in the control condition were almost equal to the physical values of *t*_S_ (**Figure 5**). The PSEs in the control and in the experimental condition were very close to each other. Underestimation of *t*_S_ that should have occurred if the systematic contrast in Experiment 3 were bilateral did not take place to any observable degree. Although we do not have sufficient data to conclude that the systematic contrast observed in Experiments 1, 2, and 3 was unilateral, the underestimation of *t*_S_ was almost negligible even in conditions in which the mechanism of contrast must have worked clearly. The observed contrast was at least very close to unilateral.

## GENERAL DISCUSSION

The purpose of the present study was to observe the overestimation of an empty time interval caused by a preceding time interval. The conditions in the present study were comparable to the conditions in which time-shrinking had been reported to take place. We had assumed that time-shrinking was a unilateral perceptual assimilation of an empty time interval to a shorter preceding time interval. One may wonder whether the potential rhythmic regularity of presented patterns may be playing a crucial role, but this idea is not supported by the fact that time-shrinking took place even when the preceding time interval and the time interval to be judged were separated in time (Sasaki et al., 2002). The assumption of “assimilation” itself is not related to any particular perceptual mechanism directly, but it can give us a wider view of the observed facts. Because perceptual assimilation and contrast often appear in the same context, we examined whether a change from the unilateral assimilation, time-shrinking, could give way to contrast when the difference between the neighboring time intervals was increased. The range of the first time interval that can cause time-shrinking has been determined systematically in previous studies, and it has been established that the illusion takes place only when the difference between the neighboring time intervals was smaller than ~100 ms. This knowledge made it possible for us to focus onto the stimulus conditions in which contrast was likely to take place. As a result, overestimation of the second of the neighboring time intervals appeared systematically.

**FIGURE 6:**
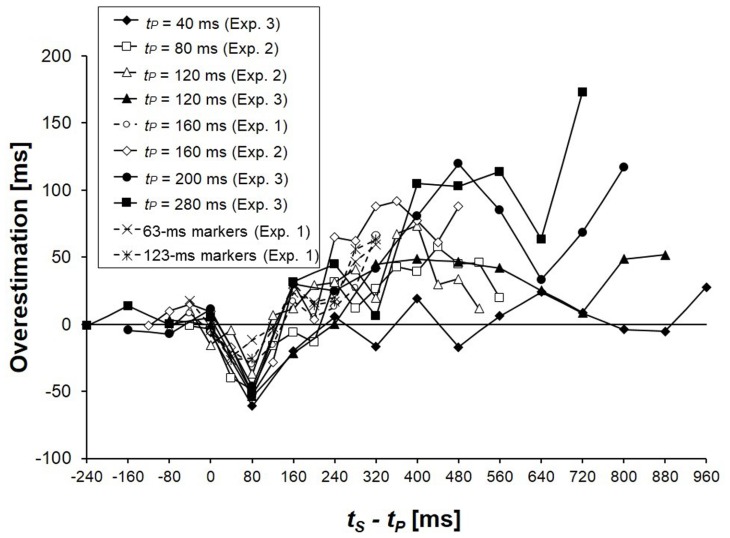
**Overestimations of *t*_**S**_ as functions of *t*_**S**_ - *t*_**P**_ in Experiments 1, 2, and 3.** The overestimations were calculated as the increases of PSEs due to the presence of *t*_P_.

When *t*_P_ precedes and neighbors *t*_S_ causing time-shrinking (i.e., the systematic underestimation of *t*_S_), an overestimation of *t*_S_ was observed when *t*_S_ was lengthened. The only exception was when *t*_P_ was set to be extremely short, i.e., *t*_P_ = 40 ms. The overestimation of *t*_S_ never disappeared when *t*_S_ - *t*_P_ > 240 ms for the other *t*_P_ values. The overestimation as a function of *t*_S_ - *t*_P_ showed a common tendency across the different *t*_P_ values (**Figure 6**), which was confirmed by the Friedman tests.

What we had not expected was that the contrast appeared in such a wide range and to such a large degree. About the range of the second time interval, we have already reached 1 s as the longest duration. It will be very important in the future to determine the upper limit of the range in which the overestimation takes place, but this would require a new experimental paradigm because we can easily reach the perceptual limit; when a time interval is equal to or above 1.5–2 s, it is often difficult to grasp the whole interval perceptually, or to perceive it as a part of a single rhythm pattern (Fraisse, 1978; Nakajima et al., 1980; Warren, 2008; see also Grondin, 2012 for a perceptual limit at around 1.5 s).

The amount of the overestimation sometimes surpassed 100 ms. Although similar overestimation had appeared occasionally in previous studies on unilateral or bilateral assimilation between neighboring time intervals, the positive overestimation had never reached 40 ms except in the present Experiment 1. It turned out now that the overestimation can be larger than time-shrinking in terms of deviation from the control PSEs in milliseconds. Although we had (re)started this study as something to be added to the studies of perceptual assimilation between time intervals, the overestimation of the second time interval now appeared as a phenomenon worth investigating more systematically in different series of studies. It is particularly necessary to examine whether the present results can be related to the fact that a successive presentation of two objects (as would be inevitable for time intervals) could facilitate the perceptual contrast between them (Ikeda and Obonai, 1955).

Fraisse (1978, 1982) argued that rhythm patterns were often based on two dominant duration values, and that they were mostly in a ratio 1:2, and occasionally in 1:3; in Western music, the shorter durations were typically 150–290 ms, and the longer durations 300–900 ms. This could explain the overestimation in the present study in some cases. Perceptual contrast can often take place as, or as a result of, categorical perception, although it is often difficult to relate results in different paradigms (Repp and Liberman, 1987). If a shorter duration and a longer duration neighboring each other are to be perceived as in different perceptual categories, i.e., in the short-duration category and in the long-duration category, this can be an aspect, or a cause, of perceptual contrast. In the present experiments, the first time interval was always below 290 ms, and the second time interval was mostly above 300 ms when it was overestimated. Most cases in which *t*_P_ caused the overestimation of *t*_S_ can be interpreted by the fact that *t*_P_ < 300 < *t*_S_ ms, which should have caused the time intervals to be relocated in different perceptual categories, which then should have led to the overestimation of *t*_S_. This interpretation describes the general tendency of the present data rather well, and is worth investigating further. However, the categorical boundary at about 300 ms is hardly a part of common knowledge, and a systematic investigation on this issue should be the first thing necessary to pursue this path.

Another possible explanation related to a categorical aspect of temporal perception is related to the studies of Miyauchi and Nakajima (2005) and ten Hoopen et al. (2006; see also Sasaki et al., 1998; and Miyauchi and Nakajima, 2007). They presented auditory temporal patterns as used in the present experiments to participants, and established a *1:1 category*, i.e., a perceptual category in which the neighboring time intervals are perceived as equal to each other even when the physical difference between them is greater than the differential limen. One of the boundaries of this category was very close to the point at which time-shrinking reaches its maximum, i.e., the point at which *t*_S_ - *t*_P_ ≃ 80 ms; the overestimation of *t*_S_ typically appeared when the difference between *t*_P_ and *t*_S_ doubled this value. This is an idea to be kept for future research, but some difficulty arises if we are to explain why the contrast appeared not immediately when the 1:1 category gave way but when the difference between *t*_P_ and *t*_S_ increased further.

Although human listeners are able to discriminate temporal patterns more precisely than specified by musical notations, they tend to establish perceptual categories represented by simple ratios between neighboring durations as in musical notations (Honing, 2013; see also Povel, 1981). It is understandable that humans have to categorize temporal patterns in order to memorize, imitate, or respond quickly to them. This might lead to the human listeners’ tendency to make the subjective ratios between neighboring durations closer to those in the prototypical patterns, which are made of simple ratios. As Fraisse (1978, 1982) indicated, the perceptual system tends to make the perceived ratio closer to a simple integral ratio as 1:1 or 1:2 (see also Honing, 2013). Supporting this observation, Nakajima (1979) reported that a pattern of two neighboring time intervals of 80 and 160 ms was perceived in ratios close to 1:1 or 1:2 avoiding intermediate cases, and Povel (1981) systematically showed the stability of the ratio 1:2 in a task to reproduce repeated temporal patterns. It is very likely that a temporal pattern to be perceived as in a ratio 1:1.7, for example, is perceptually distorted to be closer to 1:2, causing the overestimation of the second time interval. However, this alone cannot account for the overestimation observed in the present study. Suppose that *t*_P_ = 200 ms in the paradigm of Experiments 1, 2, and 3. Nakajima et al. (1988, Table 1) showed that the temporal pattern 200|400 ms was perceived in a ratio 1:1.78, i.e., closer to 1:1 than the physical ratio 1:2, and this tendency was in line with their psychophysical hypothesis. If the perceptual system tries to shift toward a simpler ratio 1:2, then the second time interval may be overestimated. Although this hypothesis seemed attractive, a further examination of our own data was not very promising. For example, in the pattern 200| 520 ms in Experiment 3, which would correspond to a subjective ratio 1:2.14 according to Nakajima et al.’s (1988) psychophysical hypothesis, the second time interval should be underestimated to make the subjective ratio closer to 1:2. In reality, this pattern still caused the overestimation of *t*_S_. As in this example, the overestimation took place more widely than was predicted from the perceptual system’s tendency toward simpler ratios. No literature or experimental data are within the present authors’ knowledge about the mechanism to show such perceptual tendencies, and the present experimental paradigm will be useful to solve this problem in the future. It should also be interesting for future research to examine the assimilation and contrast in a more complex context (e.g., Jones and McAuley, 2005).

One may wonder whether the overestimation of *t*_S_ in the present results can be explained by time-order error (TOE), which is a phenomenon observed in psychophysics in general. Previous studies reported that TOE is expected to be positive for short durations of a few hundred milliseconds, as the durations utilized as *t*_P_ in the present experiment (although it should be noted that in TOE studies two successive and distinct intervals are used instead of two intervals sharing a common marker; Woodrow, 1951; Eisler et al., 2008). This means that the duration of *t*_P_ should be overestimated relative to *t*_S._. In the present experiments, *t*_S_ was overestimated (Experiments 1–3) but *t*_P_ was not (Experiment 4). It seems difficult to explain the tendencies of the present results with TOEs as reported in classical literature (e.g., Hellström, 1985).

We began the present study in order to observe what would happen if the temporal patterns causing time-shrinking were modified by lengthening the second of the two neighboring time intervals. This tactic worked well to find clear cases in which assimilation gave way to contrast. As the overestimation was so systematic, however, it will be necessary in the future to investigate this issue in a broader paradigm apart from time-shrinking. First, it is of some interest whether the first of the neighboring time intervals is also affected when the second time interval is overestimated. The results of Experiment 4 were negative, suggesting that the contrast was unilateral, but we need further studies on this point. It attracts our interest as well whether any perceptual contrast would take place if the temporal order between the longer and the shorter time interval is reversed. Although there are some previous data for some speculation, we basically need a new set of experiments.

Arao et al. (2000) showed that time-shrinking occurred also in the visual modality, and it took place when the neighboring time intervals* t*_P_ and *t*_S_, in this order, had the relationship *t*_P_ < *t*_S_ < ~1.8 × *t*_P_. If we see their data from the present viewpoint, it is suggested that overestimation of *t*_S_ is likely to replace time-shrinking if *t*_S_ is far above this range, and this is worth investigating immediately. The same argument may hold also for the tactile modality (Hasuo et al., 2014).

One big problem for our future research is that the experimental data are not always very stable in the present paradigm, and this can be the case in other related paradigms. The individual differences were sometimes as big as the effects to be investigated. Fortunately, our present purpose was simple, i.e., to examine whether systematic overestimation of the second time interval would or would not appear; we somehow reached tentative conclusions. If the many issues suggested here are to be investigated in the future, however, we will need more sophisticated methods. One possible solution is to design experiments that enable us to perform some multivariate analyses. Another possibility is to obtain a lot of data from a few participants, and to compare results in different conditions for each individual participant.

We investigated the perception of empty time intervals marked by tone bursts, and employed temporal patterns of two neighboring time intervals. Our research question was whether the overestimation of the second time interval would replace the underestimation (time-shrinking) if the difference between the neighboring time intervals was increased. The overestimation took place very systematically when the first time interval was 80–280 ms, and its amount sometimes exceeded 100 ms, indicating that this was an important phenomenon related to rhythm perception. It is very likely that similar temporal patterns appear often in music. Assimilation and contrast, which Fraisse (1978) considered to be two important principles to construct rhythm, were manifested in an *in vitro* situation.

## Conflict of Interest Statement

The authors declare that the research was conducted in the absence of any commercial or financial relationships that could be construed as a potential conflict of interest.
